# Metabolic plasticity imparts erlotinib-resistance in pancreatic cancer by upregulating glucose-6-phosphate dehydrogenase

**DOI:** 10.1186/s40170-020-00226-5

**Published:** 2020-09-21

**Authors:** Neha Sharma, Alok Bhushan, Jun He, Gagan Kaushal, Vikas Bhardwaj

**Affiliations:** 1 Department of Pharmaceutical Sciences, Jefferson College of Pharmacy, Thomas Jefferson University, Philadelphia, PA USA; 2 Department of Pathology, Anatomy & Cell Biology, Sidney Kimmel Medical College, Thomas Jefferson University, Philadelphia, USA

**Keywords:** Erlotinib resistance, Metabolic reprogramming, Pancreatic cancer

## Abstract

Pancreatic ductal adenocarcinoma (PDAC) is one of the most malignant forms of cancer. Lack of effective treatment options and drug resistance contributes to the low survival among PDAC patients. In this study, we investigated the metabolic alterations in pancreatic cancer cells that do not respond to the EGFR inhibitor erlotinib. We selected erlotinib-resistant pancreatic cancer cells from MiaPaCa2 and AsPC1 cell lines. Metabolic profiling of erlotinib-resistant cells revealed a significant downregulation of glycolytic activity and reduced level of glycolytic metabolites compared to the sensitive cells. The resistant cells displayed elevated expression of the pentose phosphate pathway (PPP) enzymes involved in ROS regulation and nucleotide biosynthesis. The enhanced PPP elevated cellular NADPH/NADP+ ratio and protected the cells from reactive oxygen species (ROS)-induced damage. Inhibition of PPP using 6-aminonicotinamide (6AN) elevated ROS levels, induced G1 cell cycle arrest, and sensitized resistant cells to erlotinib. Genetic studies identified elevated PPP enzyme glucose-6-phosphate dehydrogenase (G6PD) as an important contributor to erlotinib resistance. Mechanistically, our data showed that upregulation of inhibitor of differentiation (ID1) regulates G6PD expression in resistant cells thus contributing to altered metabolic phenotype and reduced response to erlotinib. Together, our results highlight an underlying role of tumor metabolism in PDAC drug response and identify G6PD as a target to overcome drug resistance.

## Introduction

Epidermal growth factor receptor (EGFR), first described in the early 1980s, is a transmembrane tyrosine kinase receptor that is deregulated in various tumors [1, 2]. Upregulation or mutation of EGFR has been associated with the progression of non-small cell lung carcinoma (NSCLC), pancreatic cancers, colorectal cancers, and glioblastomas, among other tumors. EGFR-targeted therapies, including monoclonal antibodies (e.g., cetuximab) and small molecule inhibitors (e.g., erlotinib, gefitinib), have become valuable therapeutic tools. Small molecule inhibitors of EGFR have especially been beneficial for NSCLC and pancreatic ductal adenocarcinoma (PDAC) patients.

PDAC is one of the most malignant forms of cancer with an overall 5-year survival rate of 8%. With approximately 44,300 deaths in the year 2018, it is the third leading cause of cancer-related deaths in the USA [3]. Late diagnosis, lack of effective treatment options, and drug resistance make PDAC one of the most difficult cancers to treat [4, 5]. Analyses of PDAC patient samples revealed that EGFR is overexpressed in more than 40% of cases and is associated with poor disease prognosis, invasion, and aggressive clinical behavior [6–9]. Inhibition of EGFR in combination with chemo/radiation therapy has been extensively tested in pancreatic cancer patients. In patients with advanced pancreatic cancer, the addition of erlotinib to the treatment regimen improved the overall survival, progression-free survival, and disease control compared to gemcitabine alone [10]. While approximately 53% of patients in the study displayed positive EGFR expression, the presence of EGFR did not correlate with a favorable drug response [10]. Other studies have demonstrated that the presence of EGFR mutation, development of rash, and the *KRAS/TP53* mutation are predictive biomarkers for erlotinib efficacy in PDAC [11–13]. Despite positive patient response, drug resistance presents significant challenges for the continuous use of erlotinib.

Proteomic analysis of pancreatic cancer found that an altered metabolism consistent with the Warburg effect plays a vital role in PDAC progression [14–16]. Due to these modifications, the cancer cells are dependent on glycolysis for their energy production. The pentose phosphate pathway (PPP) that branches from the glycolysis plays an important role in cellular redox control and nucleotide generation, and is found to be upregulated in various tumors. The altered metabolic profile provides the proliferating cancer cells with rapid ATP synthesis and carbon for the biosynthesis of nucleotides, lipids, and proteins [17]. Recent studies have demonstrated a key role of *c-myc* in regulating pancreatic cancer metabolism. The proto-oncogene *myc* acts downstream of numerous signaling pathways such as PI3K-Akt, MEK-Erk, and Notch and induces a transcriptional response leading to tumorigenesis and cancer progression [18]. In pancreatic cancers, *c-myc* regulates global transcription of metabolic genes and induces Kras-mediated metabolic changes [19, 20]. Inhibition of *c-myc* reduced the survival of pancreatic cancers and altered cellular nucleotide pool [19, 21]. Although a few studies have attempted to evaluate the role of tumor metabolism on cancer chemotherapy resistance, its role in affecting cancer cell response to a targeted therapy such as erlotinib is not well-established [22–24].

Through the study outlined below, we characterized the metabolic alterations that underline erlotinib resistance in PDAC. Further, we investigated the therapeutic potential of targeting the metabolism of tumors that are resistant to anti-EGFR therapy.

## Results and discussion

### EGFR tyrosine kinase inhibitor (TKI) resistant cells have altered metabolic profile

**Fig. 1:**
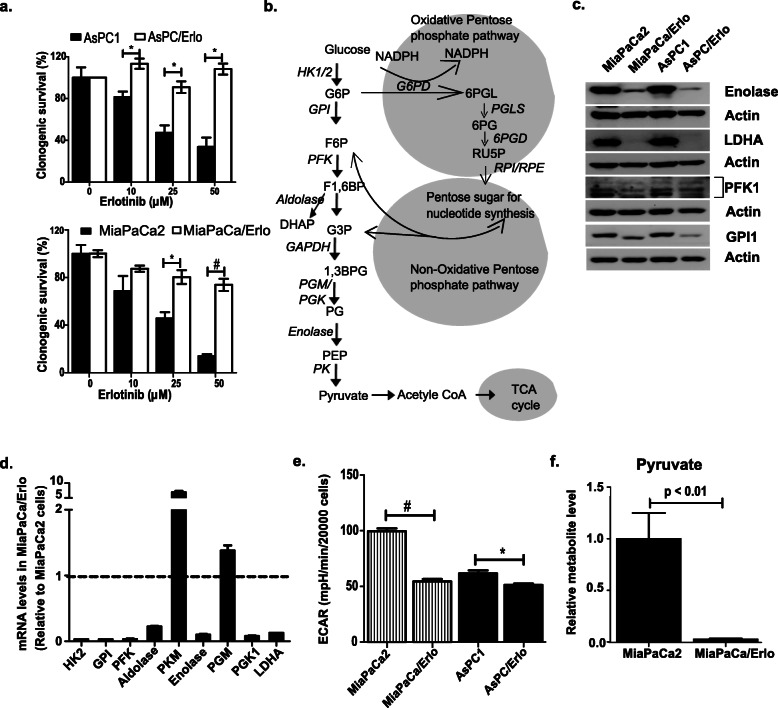
Erlotinib-resistant cells display downregulated glycolysis. **a** Effect of erlotinib treatment (48 h) on the survival of erlotinib-sensitive (MiaPaCa2 and AsPC1) and erlotinib-resistant (MiaPaCa/Erlo and AsPC/Erlo) cells was analyzed using clonogenic assay (*n* = 3). **b** Representation of central carbon metabolism. **c**, **d** Immunoblot and real-time PCR analyses depicting glycolytic enzyme levels in drug-sensitive and drug-resistant cells. HK2, hexokinase 2; GPI, glucose phosphate-isomerase; PFK, phosphofructokinase; PKM, pyruvate kinase M; PGM, phosphoglycerate mutase; PGK, phosphoglycerate kinase; LDHA, lactate dehydrogenase A (*n* = 2) (*n* = 3). **e** The metabolic phenotype was assessed in cells using Seahorse metabolic analyzer. Graph showing ECAR (glycolysis) levels in sensitive and resistant cells (*n* = 3). **f** Pyruvate levels were assessed in cells using LC-MS analysis. Graph showing pyruvate levels relative to MiaPaCa2 cells (*n* = 2). Data presented as average ± SEM (*p < 0.05, ^#^*p* < 0.01)

To understand the metabolic regulations in the cells that do not respond to EGFR-targeted therapies, we selected erlotinib-resistant cells by culturing pancreatic cancer cells MiaPaCa2 and AsPC1 in increasing concentrations of erlotinib. Since these cell lines harbor KRAS mutations, p53 mutation, and wild-type EGFR status, they represent a phenotype commonly observed in pancreatic cancers and thus serve as good model systems. The cells were cultured until stable resistant phenotypes MiaPaCa/Erlo and AsPC/Erlo were achieved. Erlotinib-resistance was assessed using MTT assays (Supplemental S1a) and confirmed by clonogenic assay (Fig. 1a and supplemental S1b). Our cell cycle analysis revealed a significant increase in S and G2/M phase population in addition to enhanced cellular proliferation (Supplemental S1c and S1d) in resistant cells. The increased proliferation observed in resistant cells was due to increased levels of cyclin D1, cyclin E, and cyclin A as demonstrated by our immunoblot analysis (Supplemental S1e). Although the increased proliferation is not associated with poor therapy response, previous reports have demonstrated that cells with cyclin D1 overexpression respond poorly to EGFR-targeted therapies [25, 26].

Investigation of signaling downstream of EGFR revealed that resistant cells exhibit reduced Erk activation, whereas no apparent effect was observed in the activation of Akt (data not shown). To interrogate the metabolic adaptations, we determined how the central carbon metabolism is altered in the resistant cells. A schematic diagram of the glycolysis channeling metabolites into the tricarboxylic acid cycle (TCA) and pentose phosphate pathway (PPP) is shown in Fig. 1b. Since the upregulation of glycolysis has been associated with reduced tumor response to various therapies, we analyzed the levels of glycolytic enzymes in our resistant cells [27]. Immunoblot and real-time PCR analysis showed that the resistant cells have reduced protein and mRNA levels of glycolytic enzymes (Fig. 1c, d, and Supplemental 2a). We then compared the metabolic phenotype of drug-sensitive and drug-resistant cells using Seahorse metabolic analyzer. We found that resistant cells displayed reduced extracellular acidification rate (ECAR, a measure of glycolysis) and elevated oxygen consumption rate (OCR, a measure of oxidative phosphorylation) (Fig. 1e and Supplemental S2b) in comparison to the erlotinib-sensitive cells. The results contrast with those of Ye et al., who reported that an acquired erlotinib-resistant model of lung cancer cells harboring mutant EGFR showed enhanced cellular dependence on glycolysis [28]. Apart from the different cancer types, the cells used in the study also differ in their EGFR status, (they used EGFR mutant cell lines) which may explain the different outcomes. The data suggest that EGFR genetic status plays a critical role in metabolic alterations observed in cancer cells. We then found that the uptake of glucose is significantly reduced in the resistant cells compared to the parent cell line (Supplemental 3a). The low uptake may account for low glycolytic activity in the resistant cells. To confirm the alteration in glycolysis, we performed liquid chromatography-tandem mass spectrometry and found that the resistant cells had lower levels of pyruvate and other key glycolytic metabolites (Fig. 1f and Supplemental S3b). The results suggest that inhibition of glycolysis may be protective of erlotinib-induced cytotoxicity. To corroborate our findings, we performed clonogenic survival assay in MiaPaCa2 cells treated with a combination of erlotinib and glycolytic inhibitor, 3-bromopyruvate (3BP, an inhibitor of enzyme hexokinase 2). Our results show that addition of 3BP induced a non-significant increase in survival of cells compared to cells treated with erlotinib alone (Supplemental S4). Our results show that the downregulation of glycolysis is associated with the poor response of pancreatic cancer cells to erlotinib.

### Erlotinib-resistant cells exhibit upregulated pentose phosphate pathway

**Fig. 2:**
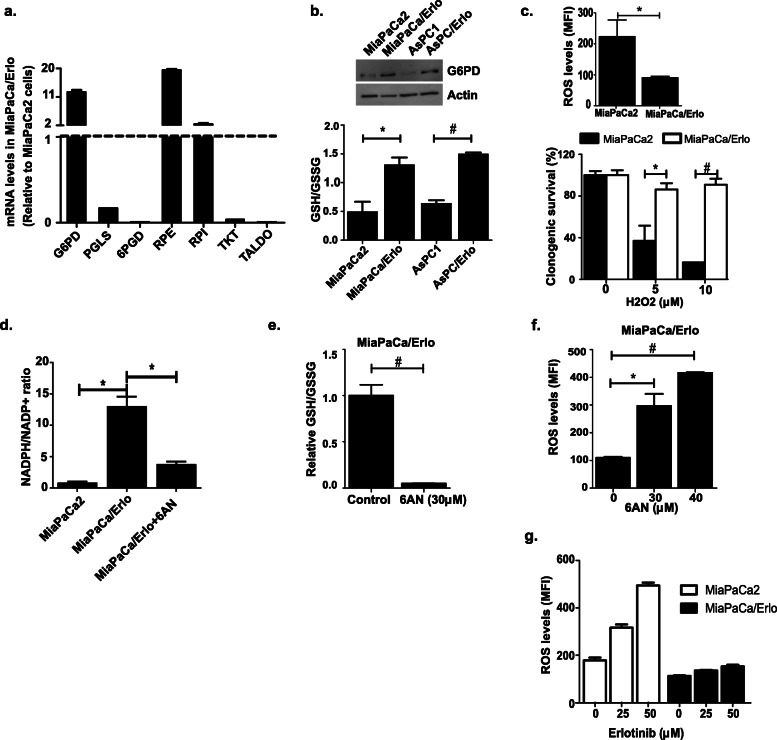
Upregulated PPP protects resistant cells from oxidative stress. **a** Real-time PCR analysis was performed to determine the levels of pentose phosphate pathway (PPP) enzymes. Graph represents enzyme levels in MiaPaCa/Erlo cells relative to MiaPaCa2 cells. G6PD, glucose 6-phoshate dehydrogenase; PGLS, 6-Phosphogluconolactonase; 6PGD, 6-phosphogluconate dehydrogenase; RPE, ribulose-phosphate 3-epimerase; RPI, ribulose-phosphate 4-isomerase; TKT, transketolase; TALDO, transaldolase (*n* = 2). **b** Drug-sensitive and drug-resistant cells were analyzed for G6PD levels (immunoblot) and glutathione content (glutathione detection kit) (*n* = 3). **c** DCFDA assay was performed to determine overall ROS levels in cells (top) (*n* = 3). Cells treated with hydrogen peroxide (H_2_O_2_) for 10 min at indicated concentration were analyzed for clonogenic survival (bottom) (*n* = 2). **d** Untreated and 6AN-treated (48 h) cells were analyzed for NADPH/NADP+ content using commercial kit (*n* = 2). **e**, **f** Effect of PPP inhibition (48 h 6AN treatment at indicated concentrations) was determined on cellular ROS and glutathione content in MiaPaCa/Erlo cells (*n* = 3). **g** Erlotinib-treated (48 h) cells were analyzed for ROS levels using DCFDA dye (*n* = 3). Data presented as average ± SEM (*p < 0.05, ^#^*p* < 0.01)

To delineate the metabolic deregulation in resistant cells, we performed real-time PCR to analyze changes in enzymes involved in the PPP. We found that the expression of PPP enzymes, ribulose-phosphate 3-epimerase (RPE), and ribulose-phosphate 4-isomerase (RPI) were significantly upregulated in the resistant cells (Fig. 2a and Supplemental S5a). As part of the non-oxidative PPP, RPE and RPI play a crucial role in nucleotide biosynthesis, and their upregulation would support the highly proliferative phenotype of resistant cells (Fig. 1b and Supplemental S1d). Our real-time PCR and immunoblot analyses also showed that the resistant cells had elevated levels of enzyme glucose-6-phosphate dehydrogenase (G6PD) (Fig. 2a, b, and Supplemental S5a). G6PD is the rate-limiting enzyme that channels glycolytic metabolite glucose-6-phosphate into the oxidative PPP. G6PD, along with 6-phosphogluconate dehydrogenase (6PGD), is a key source for reduced nicotinamide adenine dinucleotide phosphate (NADPH) in the cells. The enzyme glutathione reductase utilizes the generated NADPH to reduce glutathione (GSSG ➔ GSH) and therefore maintains the cellular redox balance. However, we found that the level of 6PGD is significantly suppressed in erlotinib-resistant cells. A recent study found that metastatic subclones of pancreatic cancers are dependent on 6PGD for their tumorigenic growth. Interestingly, the authors found that 6PGD derived its substrates from outside of PPP. These substrates (glucuronate and gluconate) successfully elevated the levels of NADPH in metastasized pancreatic cancer cells, however, failed to do so in a non-metastatic pancreatic cell line [29]. In another study, senescence induced by 6PGD knockdown was not associated with inhibition of NADPH levels [30]. This suggests that although both G6PD and 6PGD are part of oxidative PPP, their regulation and metabolical functions are independent of each other.

Consistent with elevated G6PD levels, we found that the resistant cells had an elevated GSH/GSSG ratio compared to the sensitive cells (Fig. 2b). A previous report indicated that an increase in glutathione reduced cellular ROS burden and increased ROS-detoxifying property of cancer cells [31]. Our results showed that both erlotinib-resistant cell lines had reduced overall ROS levels compared to the parent cells (Fig. 2c and Supplemental S5b). In addition, the resistant cells were comparatively less sensitive to hydrogen peroxide (H_2_O_2_)-induced cytotoxicity, indicating that the active antioxidant mechanism protects the cells from oxidative stress (Fig. 2c and Supplemental S5b).

We then confirmed if the altered PPP was responsible for the increased glutathione and lower ROS levels observed in the resistant cells. We found that treatment with oxidative PPP inhibitor 6-aminonicotinamide (6AN) reduced the cellular NADPH/NADP+ ratio and GSH content in the resistant cells leading to elevated ROS levels (Fig. 2d, e, f, Supplemental S5c, S5d, and S5e). Since G6PD is the primary NADPH provider in cancer cells [32], elevated levels of NADPH in cells with suppressed 6PGD could be attributed to enhanced G6PD level and activity. A recent study highlighted that the induction of ROS is essential for cytotoxicity of erlotinib [33]. Hence, we hypothesized that altered metabolic profile would prevent erlotinib-induced ROS generation in resistant cells and thus protect the cells from erlotinib-induced cytotoxicity. We found that treatment with erlotinib (50 μM) caused a considerable increase in ROS levels in the sensitive cells (2.7-fold relative to baseline); however, only a small increase in ROS was observed in resistant cells (1.3-fold relative to baseline) (Fig. 2g). Our results indicate that upregulation of oxidative PPP protects the resistant cells by regulating the cellular redox capacity through (1) elevated G6PD and glutathione levels and (2) lower ROS levels that protect cells against erlotinib’s cytotoxicity.

### Inhibition of the oxidative PPP targets erlotinib-resistant cells

**Fig. 3:**
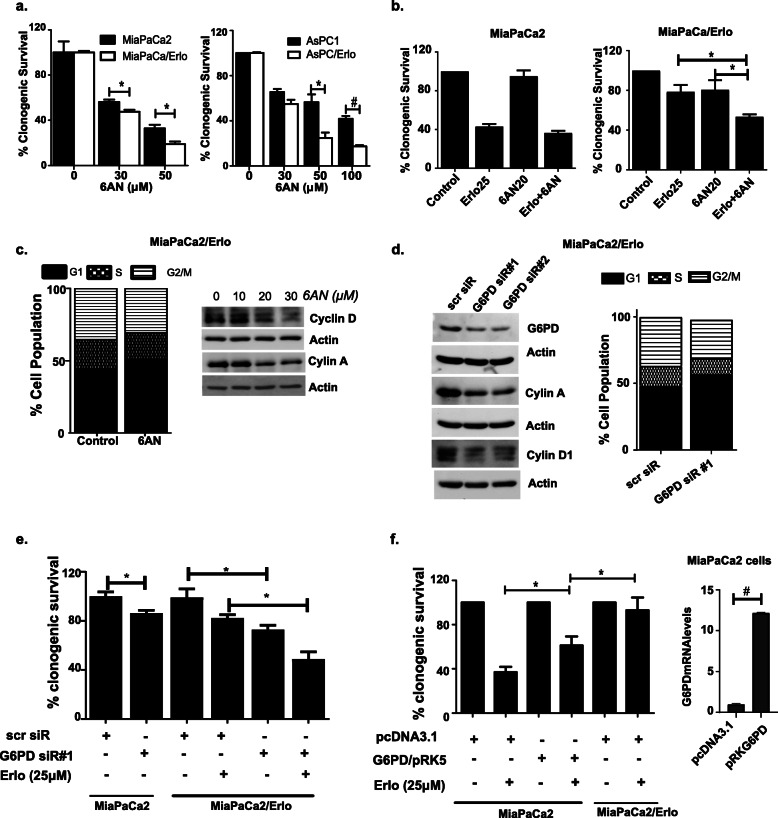
Resistant cells are sensitive to PPP inhibition. **a** Graph depicting clonogenic survival of resistant and sensitive cells treated with 6AN for 48 h at indicated concentrations (*n* = 4). **b** Clonogenic survival assay was performed on drug-sensitive and drug-resistant cells treated with erlotinib in combination with 6AN (48-h combination treatment) (*n* = 3). **c** Cell cycle analysis was performed on resistant cells treated with 6AN (48 h) by flow cytometry using propidium iodide stained cells (*n* = 4). Effect of 6AN on cyclins was ascertained using immunoblot analysis (*n* = 3). **d** Effect of G6PD knockdown (72-h post siRNA transfection) on MiaPaCa/Erlo cell cycle distribution and cyclin levels was determined (*n* = 2). **e** Effect of G6PD knockdown on MiaPaCa/Erlo cell sensitivity to erlotinib was determined using clonogenic assay. Comparative effect of G6PD siRNA on survival of MiaPaCa2 and MiaPaCa/Erlo cell was also determined in same experiment (*n* = 2). **f** MiaPaCa2 cells transfected with G6PD overexpression plasmid (G6PD/pRK5) were analyzed for their sensitivity to erlotinib using clonogenic survival assay (*n* = 3). The results were compared with empty vector transfected MiaPaCa/Erlo cells that were treated with erlotinib. Data presented as average ± SEM (*p < 0.05, ^#^*p* < 0.01)

We then hypothesized that the metabolic alterations in resistant cells would change the cell’s sensitivity to metabolic inhibitors. To test this, we performed MTT survival assay and found that compared to the sensitive cells, the resistant cells were less responsive to the cytotoxicity of a glycolytic inhibitor iodoacetic acid (IAA, an inhibitor of glycolytic enzyme glyceraldehyde 3-phosphate-dehydrogenase) (Supplemental S6a and S6b). Since the resistant cells displayed lower glycolytic activity, their reduced sensitivity to glycolytic inhibitor is on expected lines. The resistant cells were more sensitive to 6-aminonicotinamide-induced cytotoxicity. Interestingly, although erlotinib-resistant cells have upregulated oxygen consumption rate (OCR, Supplemental S2b), their sensitivity to mitochondrial complex inhibitor I, rotenone, was similar to that of erlotinib-sensitive cells (Supplemental S6a and S6b). A possible explanation for this observation is that the oxygen consumption occurs at the later stage (complex IV) of the electron transport chain, whereas, rotenone inhibits the activity of complex I. Hence, similar sensitivity of both erlotinib-sensitive and erlotinib-resistant cells to rotenone may be the result of similar level or activity of electron transport chain complex I in both cell phenotype. Using clonogenic survival assay, we confirmed the enhanced sensitivity of erlotinib-resistant cells to 6AN (Fig. 3a). We then investigated whether inhibiting PPP sensitizes the resistant cells to erlotinib. Our results clearly demonstrated that combined treatment of resistant cells with erlotinib and 6AN more effectively reduced the survival of the resistant cells compared to each drug’s individual effect (Fig. 3b and Supplemental S7a). Notably, the sensitivity of parent cell lines to erlotinib was not altered by 6AN suggesting that the parent cells are less dependent on oxidative PPP for their survival (Fig. 3b and Supplemental S7a). We then expanded our observations in PANC1 cells that are inherently resistant to erlotinib. We found that treatment with 6AN enhanced the cytotoxicity of erlotinib in PANC1 cells (Supplemental S7b). The results highlight a potentially important role of PPP in cells that are inherently resistant to the drug. Cell cycle analysis revealed that treatment with 6AN leads to the accumulation of cells in the G1 phase and reduced levels of cyclin D1 (Fig. 3c and supplemental S7c). The observations were consistent with a previous report that showed 6AN halts the cell cycle progression and leads to the accumulation of cells in G1 phase [34]. We then determined the metabolic effect of PPP inhibition. Metabolic analysis found that acute treatment with 6AN induced a significant increase in the ECAR in erlotinib-resistant cells (1.9-fold), whereas the increase in drug-sensitive cells was less prominent (1.3-fold) (Supplemental S7d). With suppressed glycolysis associated with reduced erlotinib cytotoxicity, upregulation of glycolysis by 6AN treatment may represent a metabolic phenotype that responds well to erlotinib.

Although 6AN targets two PPP enzymes—G6PD and 6PGD, levels of 6-phosphogluconate dehydrogenase were not increased in the resistant cells. Therefore, we hypothesized that the selectivity of 6AN’s cytotoxic effect on resistant cells is primarily due to its inhibitory effect on G6PD, and inhibition of G6PD would sensitize the resistant cells to erlotinib. To test our hypothesis, we silenced G6PD using siRNA and found accumulation of cells in G1 phase and downregulation of cyclin D1 and cyclin A levels in erlotinib-resistant cells (Fig. 3d and Supplemental S8a). siRNA-mediated inhibition of G6PD has previously shown to reduce cyclin D1 levels [35]. We then determined how inhibition of G6PD alters the sensitivity of resistant cells to erlotinib. Our results clearly show that G6PD downregulation enhanced erlotinib’s cytotoxicity in the resistant cells (Fig. 3e and Supplemental S8b). We also found that inhibition of G6PD reduced the survival of drug-sensitive cells (Fig. 3e). Although the difference in survival of parent and erlotinib-resistant cells treated with G6PD siRNA was relatively small, we consistently observed enhanced cytotoxicity of G6PD downregulation on the resistant cells. Our results clearly indicate that the elevated G6PD in erlotinib-resistant cells plays a critical role in their survival and blunting the response of erlotinib. The results also highlight that G6PD plays an important role in the survival of erlotinib-sensitive pancreatic cancer cells, which considering the importance of G6PD as an essential NADPH generator are not surprising.

To further confirm the role of G6PD in promoting erlotinib resistance, we transiently overexpressed G6PD in pancreatic cancer cells and determined their response to erlotinib. Although G6PD overexpression significantly reduced the sensitivity of cells to erlotinib, the extent of protection did not completely recapitulate as observed in the resistant cells (Fig. 3f and Supplemental S8c). This outcome strongly suggests that mechanism alternative to G6PD overexpression could also contribute to erlotinib resistance.

### C-myc regulates the metabolic reprogramming of resistant cells

**Fig. 4:**
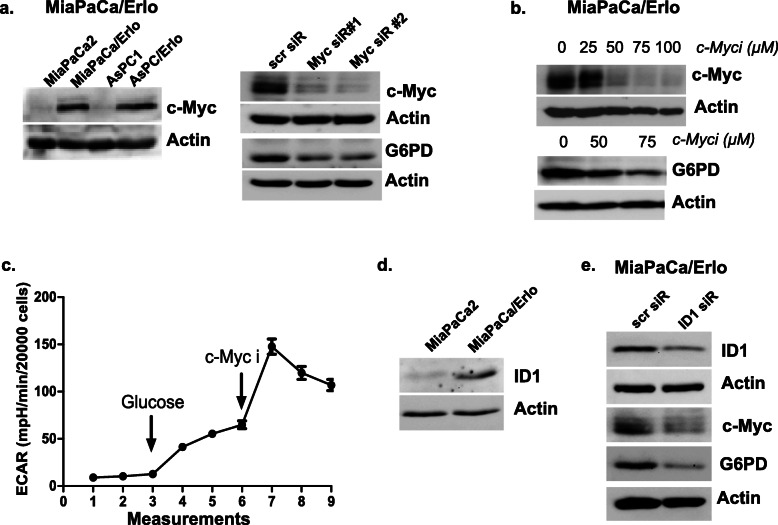
Myc regulates G6PD levels in resistant cells. **a** Immunoblot depicting c-myc levels in the resistant cells. Effect of siRNA-mediated downregulation of c-myc on G6PD levels was assessed using immunoblot analysis (*n* = 3). **b** Effect of c-myc inhibition, *c-myci* (10058F4, c-myc inhibitor), was analyzed on G6PD levels (*n* = 3). **c** Effect of acute *c-myci* treatment on extracellular acidification rate of MiaPaCa/Erlo cells was analyzed using Seahorse metabolic analyzer (*n* = 2). **d** Levels of ID1 were compared in drug-sensitive and drug-resistant cells using immunoblot analysis (*n* = 3). **e** Effect of ID1 knockdown was assessed on levels of c-myc and G6PD in MiaPaCa/Erlo cells using immunoblot analysis (*n* = 3)

To delineate the underlying mechanism for metabolic reprogramming, we performed immunoblot analysis and found that resistant cells expressed higher levels of *c-myc* compared to sensitive cells (Fig. 4a). Prior studies have demonstrated that *c-myc* plays a central role in tumor cell’s metabolic reprogramming [36]. We found that inhibiting c-myc through siRNA suppressed the levels of G6PD in the resistant cells (Fig. 4b). MYC interacts with MAX leading to E-box-dependent transcription of c-myc-induced genes. Using an inhibitor of MYC-MAX interaction, 10058-F4, we found that inhibition of c-myc decreased G6PD levels in the resistant cells (Fig. 4b). The results confirm that c-myc regulates G6PD levels in the resistant cells and plays a central role in upregulation of PPP. To determine the metabolic effect of c-myc inhibition, we performed seahorse analysis and found that acute treatment of c-myc inhibitor enhanced the glycolytic rate (ECAR) in the resistant cells (Fig. 4c). Our results provide key mechanistic insight on how c-myc controls the metabolic alterations in the erlotinib-resistant cells wherein c-myc promotes the oxidative pentose phosphate pathway through upregulation of G6PD. Consistent with our findings, recent reports demonstrated that c-myc plays a role in G6PD transcription through multiple mechanisms [37, 38]. To uncover the underlying mechanism for c-myc upregulation in resistant cells, we performed immunoblot analysis and found that resistant cells expressed high levels of inhibitor of differentiation 1 (ID1) (Fig. 4d). ID1 is a member of helix-loop-helix (HLH) family of proteins that act as an oncogene. A recent report highlighted that ID1 regulates the levels of c-myc and G6PD in oxaliplatin-resistant hepatocellular carcinoma cells, thus activating the PPP [37]. Using ID1-specific siRNA, we found that inhibition of ID1 suppressed the levels of c-myc and G6PD in erlotinib resistant cells (Fig. 4e). Our results suggest that upregulation of ID1 supports the altered phenotype in erlotinib-resistant cells by activating the ID1-c-myc-G6PD axis.

## Conclusion

Overall, our novel findings reveal that pancreatic cancer cells resistant to erlotinib have an altered metabolic profile, and these alterations could potentially be targeted to overcome drug resistance. The altered metabolism is characterized by reduced glycolytic activity in the resistant cells. The upregulation of PPP represents a necessary adaptation that allows the cancer cells to increase their proliferation and keep cellular ROS levels low. The enhanced vulnerability of the resistant cells to metabolic inhibitor such as 6AN presents an attractive strategy to target cancer cells resistant to erlotinib.

## Materials and methods

### Cell culture

Human pancreatic carcinoma cell lines AsPC1 (CRL-1682), MiaPaCa2 (CRL-1420), PANC-1 (CRL-1469), and BxPC-3 (CRL-1687) were obtained from American Type Culture Collection (Manassas VA) and maintained in Dulbecco’s modified Eagle’s medium supplemented with fetal bovine serum and antibiotics in a humidified atmosphere with 5% CO_2_ at 37 °C. Cell lines were characterized and authenticated by the supplier.

### Generation of stable resistant cell lines (erlotinib-resistant cell lines)

AsPC1 and MiaPaCa2 parental cell lines were treated with increasing concentration of erlotinib HCl [OSI-744] (Selleckchem, Houston, TX) until stable resistant cell lines were obtained*.* Cells were cultured in drug-free media for 4–5 days before experiments to prevent acute drug effects.

### Drugs, siRNAs, transfection, and plasmid

G6PD/pRK5 was a gift from Xiaolu Yang (Addgene plasmid # 41521). Plasmid transfection was carried out using Lipofectamine 2000 (Invitrogen, Carlsbad, CA). siRNA directed against human c-myc was purchased from Invitrogen (Stealth siRNA) (c-myc siRNA #1: GCGGAAACGACGAGAACAGUUGAAA/UUUCAACUGUUCUGUCGUUUCCGC) and Integrated DNA technology (IDT, Coralville, Iowa) (c-myc siRNA# 2: CAAACUUGAACAGCUACGGAACUCT, AGAGUUCCGUAGCUGUUCAAGUUUGUG) and transfected using Lipofectamine RNAiMax (Invitrogen). Predesigned siRNAs for G6PD (G6PD siRNA #1, G6PD siRNA #2, and ID1 siRNA) were purchased from IDT. G6PD inhibitor 6-aminonicotinamide (6AN) and c-myc inhibitor 10058-F4 were purchased from Selleckchem. 3-Bromopyruvate was purchased from Sigma-Aldrich (St. Louis, MO).

### MTT survival assay

MTT survival assay was performed as mentioned previously [39]. Briefly, treated cells were incubated with 20 μL of MTT reagent (3-(4, 5-dimethylthiazolyl-2)-2, 5-diphenyltetrazolium bromide) made in 1X PBS at 5 mg/ml for 4 h, and formazan crystals were dissolved in DMSO to determine cell survival.

### Clonogenic survival assay

Parental (2000 cells/well) and resistant (1000 cells/well) cells were plated in a 6-well plate and allowed to attach overnight. The cells were then treated with the mentioned drugs for indicated times and allowed to grow drug-free for 7 days. Colonies were stained with 5% crystal violet stain in 80% methanol. Image J was used to calculate the staining intensity (ImageJ.nih.gov).

### Immunoblot analysis

Immunoblot assays were performed as mentioned previously [39]. BioRad DC assay was used to determine protein content. β-Actin was used as the loading control. Information for antibodies can be found in supplementary information SI1.

### Cell cycle analysis

Treated cells were collected and washed with cold 1XPBS. Cells were fixed using ice-cold 80% ethanol solution overnight at − 20 °C. The cells were then stained with propidium iodide (PI) (50 μg/ml) in the presence of RNase A (100 μg/ml) and analyzed using flow cytometry (Guava EasyCyte, Millipore Sigma, Burlington, MA).

### RNA extraction and real-time polymerase chain reaction

Total RNA was extracted using the RNAeasy kit (Qiagen, Germantown, MD) or triazole (Invitrogen) according to the manufacturer’s recommendations. RNA content was quantified using nanodrop, and 2 μg of RNA was reverse transcribed using the High Capacity cDNA Reverse Transcription Kit (Applied Biosystems, Foster City, CA) as per the manufacturer’s protocol. Quantitative RT-PCR was performed using the Quant3 Studio (Invitrogen) using specific mRNA primers (IDT). Primer sequences are available in supplementary information SI1. HPRT (hypoxanthine guanine phosphoribosyl transferase) was used as the reference gene.

### Reactive oxygen species assay

Cellular ROS was determined using 2′, 7′-dichlorofluorescin diacetate (DCFDA) (Sigma-Aldrich) according to the manufacturer’s protocol. Briefly, cells (treated as indicated) were incubated with 10 μM DCFDA dye in serum-free media for 30 min at 37 °C, washed with PBS, and analyzed using flow cytometry.

### Glucose uptake assay

Cellular glucose uptake was determined using 2-deoxy-2-[(7-nitro-2,1,3-benzoxadiazol-4-yl)amino]-D-glucose (2NBDG) dye (Sigma-Aldrich) according to manufacturer’s protocol. Briefly, cells were incubated with 20 μM 2NBDG in glucose-free media for 30 min at 37 °C, washed with PBS, and analyzed using flow cytometry.

### Seahorse metabolic assay

Cellular metabolic analyses were performed using Seahorse metabolic analyzer (Agilent, Santa Clara, CA). Cellular phenotypic and glycolysis stress tests were performed according to the manufacturer’s recommendations. 15,000–20,000 cells were plated depending on the cell line. Data was normalized to protein content. Acute treatment for G6PD and c-myc inhibitors were carried out using customized protocol within glycolysis stress test according to the manufacturer’s recommendations.

### GSH/GSSG and NADPH/NADP+ measurement

Measurement of cellular glutathione content (GSH and GSSG, Glutathione Colorimetric Detection Kit, Thermo Fisher, Waltham, MA) and NADPH/NADP+ levels (NADP/NADPH Colorimetric kit, Biovision, Milpitas, CA) was performed following the manufacturer’s recommendations.

### Metabolite analyses using mass spectrometry

MiaPaCa2 and MiaPaCa/Erlo cells cultured in 100-mm dishes were washed twice with 1X PBS and collected following trypsinization. The metabolites were collected using 80% cold methanol followed by centrifugation at 14,000×*g* for 10 min at 4 °C. The supernatant was collected and dried in a SpeedVac at 30 °C. The extract was dissolved in 200 μl of MS-grade water by vortexing on ice for 10 min and centrifuged at 14,000×*g* for 10 min. The supernatant was then subjected to liquid chromatography-tandem mass spectrometry (LC-MS) analyses.

Liquid chromatography (LC) analysis was performed using a Dionex Ultimate 3000 HPLC system (Thermo Fisher) attached with Thermo Orbitrap mass spectrometer using the XBridge®Amide 3.5 μm, 4.6 × 100 mm (Waters®, Milforfd, MA) column. The mobile phase consisted of 10 mM ammonium acetate buffer (pH 9.3) and acetonitrile (50:50, %v/v). The injection volume was 5 μl, with a flow rate of 0.20 ml/min. The column temperature was maintained at 25 °C. The data acquisition from mass spectrometry was delayed for 1 min followed by 5 min of data acquisition for a total of 6 min run time. The analysis was performed under the negative ionization monitoring mode with a heated ion transfer capillary. The Exactive (v.1.1SP6) software was used for mass spectrometry method development and data acquisition. Thermo Xcalibur (v. 3.0.63) was used for integration of Chromeleon, and Exactive was used for sample injections and LC-MS data was acquired in .raw file format.

### Mass spectrometry conditions

A scanning mass range of 160–340 m/z was used for all samples. All the scans were performed under negative ion mode, and ionization was achieved using electron spray ionization. The sheath gas flow rate was maintained at 20 psi, auxiliary gas flow rate was maintained at 5 psi, electron spray voltage used for the ionization was kept at 3.5 kV, and the capillary temperature was set to 375 °C. Capillary, tube lens, and skimmer voltages were kept at − 47.50 V, − 105.0 V, and − 22 V, respectively. These parameters were selected and saved as direct infusion method for using the Thermo Exactive (v. 1.1 Sp6) software of Thermo Exactive Orbitrap Mass spec. This method was integrated with HPLC to create a single method for LC-MS runs. The metabolite levels were determined by comparing them with reference peaks obtained from metabolite standards (Sigma-Aldrich), and relative peak area was determined.

### Statistical analysis

Student’s *t* test was used to analyze statistical significance between two groups. The difference was considered significant if *p* < 0.05. Results are expressed as average ± SEM if not specifically indicated.

## Supplementary information

**Additional file 1.** Supplemental S1: (a) MTT analysis were performed to determine the effect of erlotinib on drug-sensitive and resistant cells (n= 3). (b) Representative clonogenic survival assay images are shown corresponding to Figure 1a. (c) Cell cycle analysis of erlotinib-sensitive and -resistant cells performed on propidium iodide stained cells (n= 2). (d) 15 000 cells were plated for indicated cell lines and cellular proliferation was assessed using cell count assay (n= 3). (e) Levels of cyclins were determined in sensitive and resistant cells using immunoblot analysis (n = 3). Data presented as average ± SEM (*, p < 0.05, #, p < 0.01).

**Additional file 2.** Supplemental S2: (a) Real-time PCR analysis depicting altered glycolytic enzyme mRNA levels in AsPC/Erlo cells (n= 2). (b) Oxygen consumption rate was analyzed by phenotypic assay using Seahorse Metabolic analyzer (n= 3). Data presented as average ± SEM (#, p < 0.01).

**Additional file 3.** Supplemental S3: (a) Cell labeled with 2-NBDG were analyzed for glucose uptake using flow cytometric analysis (n= 4). (b) Graph represents glycolytic metabolite levels in MiaPaCa2 and MiaPaCa/Erlo cells as measured by liquid chromatography-tandem mass spectroscopy. G6P, glucose 6-phosphate; G3P, glyceraldehyde 3-phosphate; 3PG, 3-phosphoglycerate; PEP, phosphoenolpyruvate. Metabolite level is presented as relative to MiaPaCa2 cells (n= 2). Data presented as average ± SEM (*, p < 0.05).

**Additional file 4.** Supplemental S4: MiaPaCa2 cells treated with indicated concentration of erlotinib (Erlo) and 3-bromopyruvate (3BP) for 48 hours were analyzed for clonogenic survival (n=2).

**Additional file 5.** Supplemental S5: (a) Graph representing altered pentose phosphate pathway (PPP) enzyme mRNA levels in AsPC/Erlo cells as measured by real-time PCR analysis (n =3). (b) DCFDA stained cells were used to determine ROS levels in the cells (left). Cells treated with hydrogen peroxide (30 μM) for 10 minutes were analyzed for clonogenic survival (right) (n=3). (c) Reduced (GSH) and oxidized (GSSG) glutathione levels were analyzed using glutathione assay kit. Graph representing relative GSH/GSSG content in cells treated with 6AN for 48 hours (n= 3). (d) NADPH/NADP levels were analyzed in indicated cells using commercial kit (n= 2). (e) The effect of 6AN on the induction of ROS was determined using DCFDA stained AsPC/Erlo cells (n= 4). Data presented as average ± SEM (*, p < 0.05, #, p < 0.01).

**Additional file 6.** Supplemental S6: MTT assays were performed to determine the sensitivity of (a) MiaPaCa2 and MiaPaCa/Erlo, and (b) AsPC1 and AsPC/Erlo cells to metabolic inhibitors to pentose phosphate pathway (6AN), oxidative phosphorylation (Rotenone) and glycolysis (Iodoacetic acid). Data presented as average ± SEM (n= 3) (*, p < 0.05, #, p < 0.01).

**Additional file 7.** Supplemental S7: (a) Effect of 6AN on sensitivity of AsPC1 and AsPC/Erlo cells to erlotinib (Erlo) was determined using clonogenic assay (n= 3). (b) Graph depicting sensitivity of pancreatic cancer cell lines (PANC-1, MiaPaCa2, AsPC1, and BxPC-3) to erlotinib (72-hour treatment) as measured by MTT assay (left). The effect of 6AN (48-hour treatment) on cytotoxicity of Erlotinib on PANC-1 cells was measured using clonogenic survival assay (n= 3). (c) Effect of 6AN on cell cycle was determined using propidium iodide stained cells (left). Immunoblot analysis were performed to determine alteration in cyclin levels by 48-hour 6AN treatment (right) (n= 4). (d) Effect of acute 6AN treatment (30 uM) on extracellular acidification rate of MiaPaCa2 and MiaPaCa/Erlo cells was assessed using Seahorse metabolic analyzer (n= 3). Data presented as average ± SEM (*, p < 0.05, #, p < 0.01).

**Additional file 8.** Supplemental S8: (a) Effect of G6PD knockdown (72 hours post siRNA transfection) on AsPC/Erlo cell cycle distribution was determined using flow cytometry (n =2). (b) Effect of G6PD knockdown on AsPC/Erlo cell sensitivity to erlotinib was determined using clonogenic assay (n= 3). (c) AsPC1 cells transfected with G6PD overexpression plasmid (G6PD/pRK5) were analyzed for their sensitivity to erlotinib using clonogenic survival assay. The results were compared with empty vector transfected AsPC/Erlo cells treated with erlotinib (n= 3). Data presented as average ± SEM (*, p < 0.05, #, p < 0.01.

**Additional file 9.** Supplementary Information 1.

## Data Availability

The datasets used are available from the corresponding author upon reasonable request.

## Abbreviations

EGFR: Epidermal growth factor receptor
PDAC: Pancreatic ductal adenocarcinoma
PPP: Pentose phosphate pathway
TCA: Tricarboxylic acid cycle
ECAR: Extracellular acidification rate (measure of glycolysis)
OCR: Oxygen consumption rate (measure of oxidative phosphorylation)
G6PD: Glucose-6-phosphate dehydrogenase
RPE: Ribulose-phosphate 3-epimerase
RPI: Ribulose-phosphate 4-isomerase
PGLS: 6-Phosphogluconolactonase
6PGD: 6-Phosphogluconate dehydrogenase
TKT: Transketolase
TALDO: Transaldolase
HK2: Hexokinase 2
GPI: Glucose phosphate-isomerase
PFK: Phosphofructokinase
PKM: Pyruvate kinase M
PGM: Phosphoglycerate mutase
PGK: Phosphoglycerate kinase
LDHA: Lactate dehydrogenase A
6AN: 6-Aminonicotinamide
ROS: Reactive oxygen species
DCFDA: 2′, 7′-Dichlorofluorescin diacetate (ROS dye)
GSH/GSSG: Reduced glutathione/oxidized glutathione ratio
RT-PCR: Real-time polymerase chain reaction
LCMS: Liquid chromatography mass spectrometry
