# Supplementary material for: Metabolic plasticity imparts erlotinib-resistance in pancreatic cancer by upregulating glucose-6-phosphate dehydrogenase
Source: Cancer Metab. 2020 Sep 21;8:19. doi: 10.1186/s40170-020-00226-5 (PMC7507640; doi:10.1186/s40170-020-00226-5)
Supplement: Supplementary file 1 — Additional file 1. Supplemental S1: (a) MTT analysis were performed to determine the effect of erlotinib on drug-sensitive and resistant cells (n= 3). (b) Representative clonogenic survival assay images are shown corresponding to Figure 1a. (c) Cell cycle analysis of erlotinib-sensitive and -resistant cells performed on propidium iodide stained cells (n= 2). (d) 15 000 cells were plated for indicated cell lines and cellular proliferation was assessed using cell count assay (n= 3). (e) Levels of cyclins were determined in sensitive and resistant cells using immunoblot analysis (n = 3). Data presented as average ± SEM (*, p < 0.05, #, p < 0.01). [file 40170_2020_226_MOESM1_ESM.pdf]

Supplemental S1

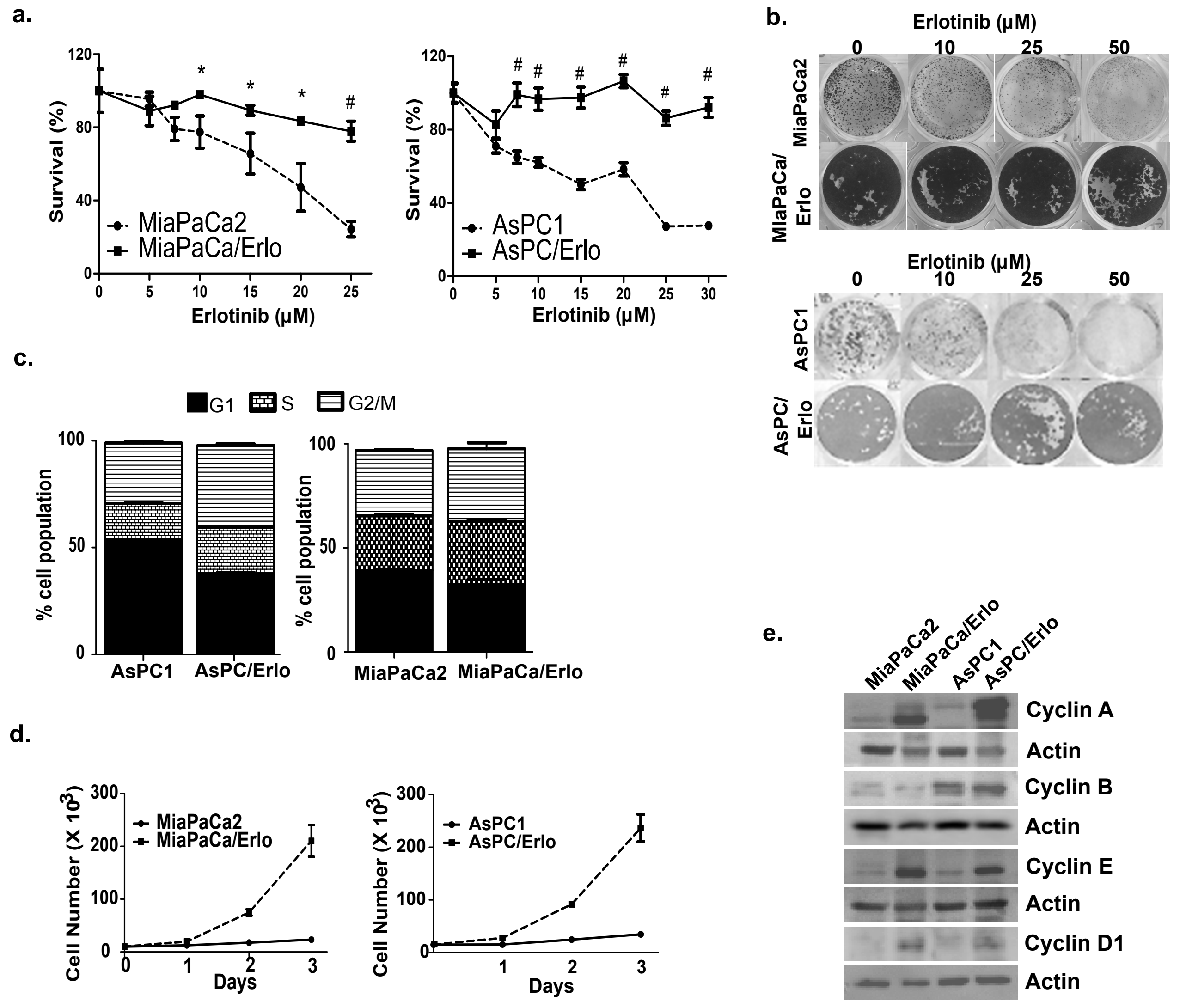

Supplemental S1: (a) MTT analysis were performed to determine the effect of erlotinib on drug-sensitive and resistant cells (n= 3). (b) Representative clonogenic survival assay images are shown corresponding to figure 1a. (c) Cell cycle analysis of erlotinib-sensitive and -resistant cells performed on propidium iodide stained cells (n= 2). (d) 15 000 cells were plated for indicated cell lines and cellular proliferation was assessed using cell count assay (n= 3). (e) Levels of cyclins were determined in sensitive and resistant cells using immunoblot analysis (n = 3). Data presented as average ± SEM (*, $p < 0.05$, #, $p < 0.01$).
