# Supplementary material for: Metabolic plasticity imparts erlotinib-resistance in pancreatic cancer by upregulating glucose-6-phosphate dehydrogenase
Source: Cancer Metab. 2020 Sep 21;8:19. doi: 10.1186/s40170-020-00226-5 (PMC7507640; doi:10.1186/s40170-020-00226-5)
Supplement: Supplementary file 2 — Additional file 2. Supplemental S2: (a) Real-time PCR analysis depicting altered glycolytic enzyme mRNA levels in AsPC/Erlo cells (n= 2). (b) Oxygen consumption rate was analyzed by phenotypic assay using Seahorse Metabolic analyzer (n= 3). Data presented as average ± SEM (#, p < 0.01). [file 40170_2020_226_MOESM2_ESM.pdf]

Supplemental S2

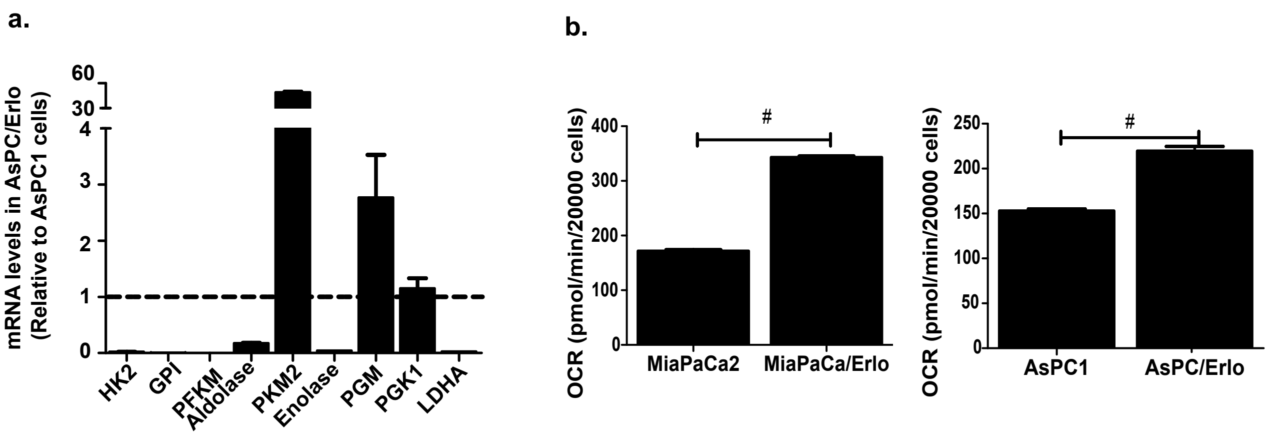

Supplemental S2: (a) Real-time PCR analysis depicting altered glycolytic enzyme mRNA levels in AsPC/Erlo cells (n= 2). (b) Oxygen consumption rate was analyzed by phenotypic assay using Seahorse Metabolic analyzer (n= 3). Data presented as average ± SEM (#, $p < 0.01$).
