# Supplementary material for: Metabolic plasticity imparts erlotinib-resistance in pancreatic cancer by upregulating glucose-6-phosphate dehydrogenase
Source: Cancer Metab. 2020 Sep 21;8:19. doi: 10.1186/s40170-020-00226-5 (PMC7507640; doi:10.1186/s40170-020-00226-5)
Supplement: Supplementary file 3 — Additional file 3. Supplemental S3: (a) Cell labeled with 2-NBDG were analyzed for glucose uptake using flow cytometric analysis (n= 4). (b) Graph represents glycolytic metabolite levels in MiaPaCa2 and MiaPaCa/Erlo cells as measured by liquid chromatography-tandem mass spectroscopy. G6P, glucose 6-phosphate; G3P, glyceraldehyde 3-phosphate; 3PG, 3-phosphoglycerate; PEP, phosphoenolpyruvate. Metabolite level is presented as relative to MiaPaCa2 cells (n= 2). Data presented as average ± SEM (*, p < 0.05). [file 40170_2020_226_MOESM3_ESM.pdf]

Supplemetal S3

a.

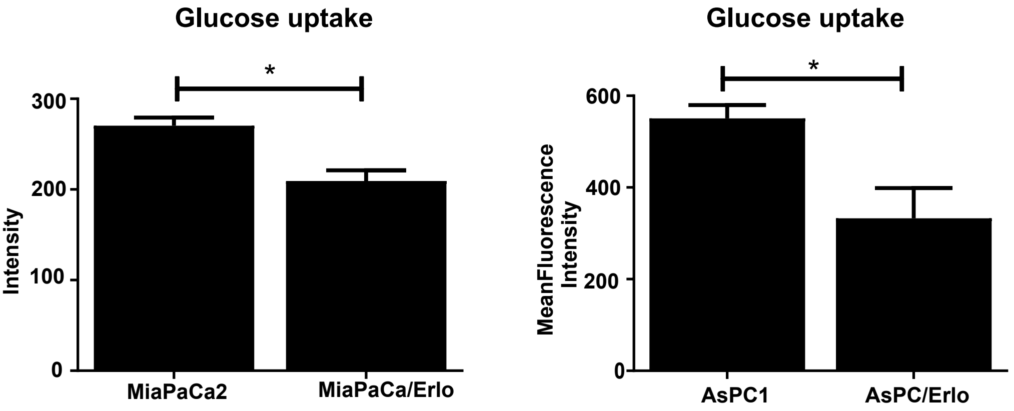

b.

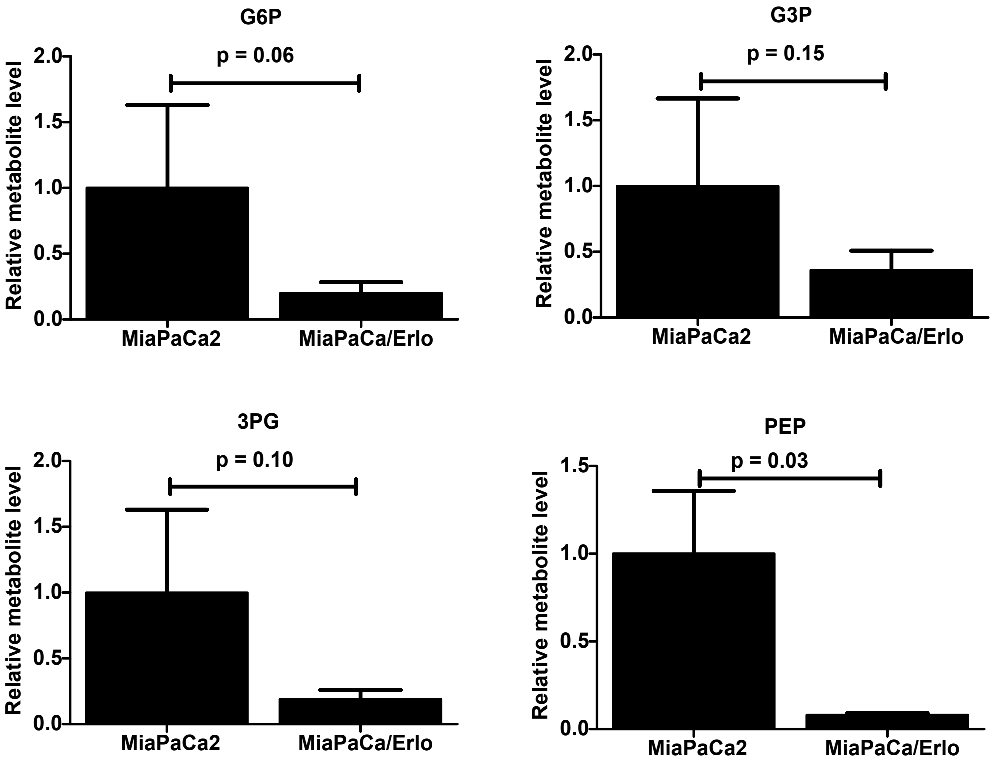

Supplemental S3: (a) Cell labeled with 2-NBDG were analyzed for glucose uptake using flow cytometric analysis (n= 4). (b) Graph represents glycolytic metabolite levels in MiaPaCa2 and MiaPaCa/Erlo cells as measured by liquid chromatography-tandem mass spectroscopy. G6P, glucose 6-phosphate; G3P, glyceraldehyde 3-phosphate; 3PG, 3-phosphoglycerate; PEP, phosphoenolpyruvate. Metabolite level is presented as relative to MiaPaCa2 cells (n= 2). Data presented as average ± SEM (*, $p < 0.05$).
