# Supplementary material for: Metabolic plasticity imparts erlotinib-resistance in pancreatic cancer by upregulating glucose-6-phosphate dehydrogenase
Source: Cancer Metab. 2020 Sep 21;8:19. doi: 10.1186/s40170-020-00226-5 (PMC7507640; doi:10.1186/s40170-020-00226-5)
Supplement: Supplementary file 4 — Additional file 4. Supplemental S4: MiaPaCa2 cells treated with indicated concentration of erlotinib (Erlo) and 3-bromopyruvate (3BP) for 48 hours were analyzed for clonogenic survival (n=2). [file 40170_2020_226_MOESM4_ESM.pdf]

Supplemetal S4

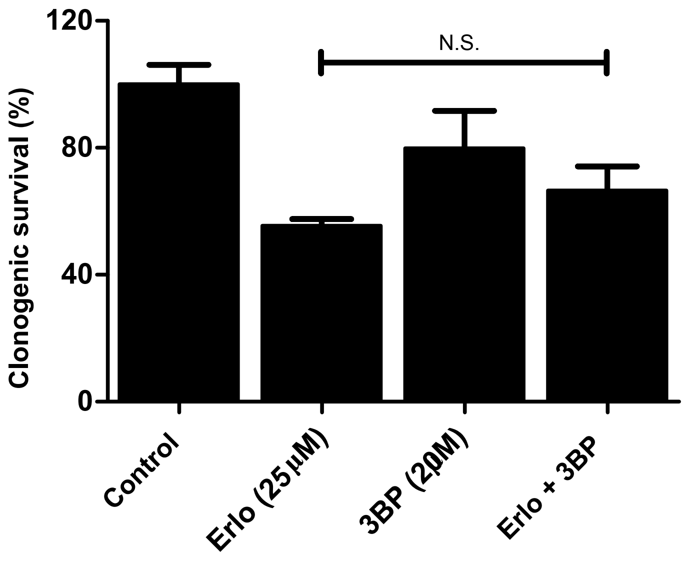

Supplemental S4: MiaPaCa2 cells treated with indicated concentration of erlotinib (Erlo) and 3-bromopyruvate (3BP) for 48 hours were analyzed for clonogenic survival (n=2).
