# Supplementary material for: Metabolic plasticity imparts erlotinib-resistance in pancreatic cancer by upregulating glucose-6-phosphate dehydrogenase
Source: Cancer Metab. 2020 Sep 21;8:19. doi: 10.1186/s40170-020-00226-5 (PMC7507640; doi:10.1186/s40170-020-00226-5)
Supplement: Supplementary file 5 — Additional file 5. Supplemental S5: (a) Graph representing altered pentose phosphate pathway (PPP) enzyme mRNA levels in AsPC/Erlo cells as measured by real-time PCR analysis (n =3). (b) DCFDA stained cells were used to determine ROS levels in the cells (left). Cells treated with hydrogen peroxide (30 μM) for 10 minutes were analyzed for clonogenic survival (right) (n=3). (c) Reduced (GSH) and oxidized (GSSG) glutathione levels were analyzed using glutathione assay kit. Graph representing relative GSH/GSSG content in cells treated with 6AN for 48 hours (n= 3). (d) NADPH/NADP levels were analyzed in indicated cells using commercial kit (n= 2). (e) The effect of 6AN on the induction of ROS was determined using DCFDA stained AsPC/Erlo cells (n= 4). Data presented as average ± SEM (*, p < 0.05, #, p < 0.01). [file 40170_2020_226_MOESM5_ESM.pdf]

**Supplemental S5**

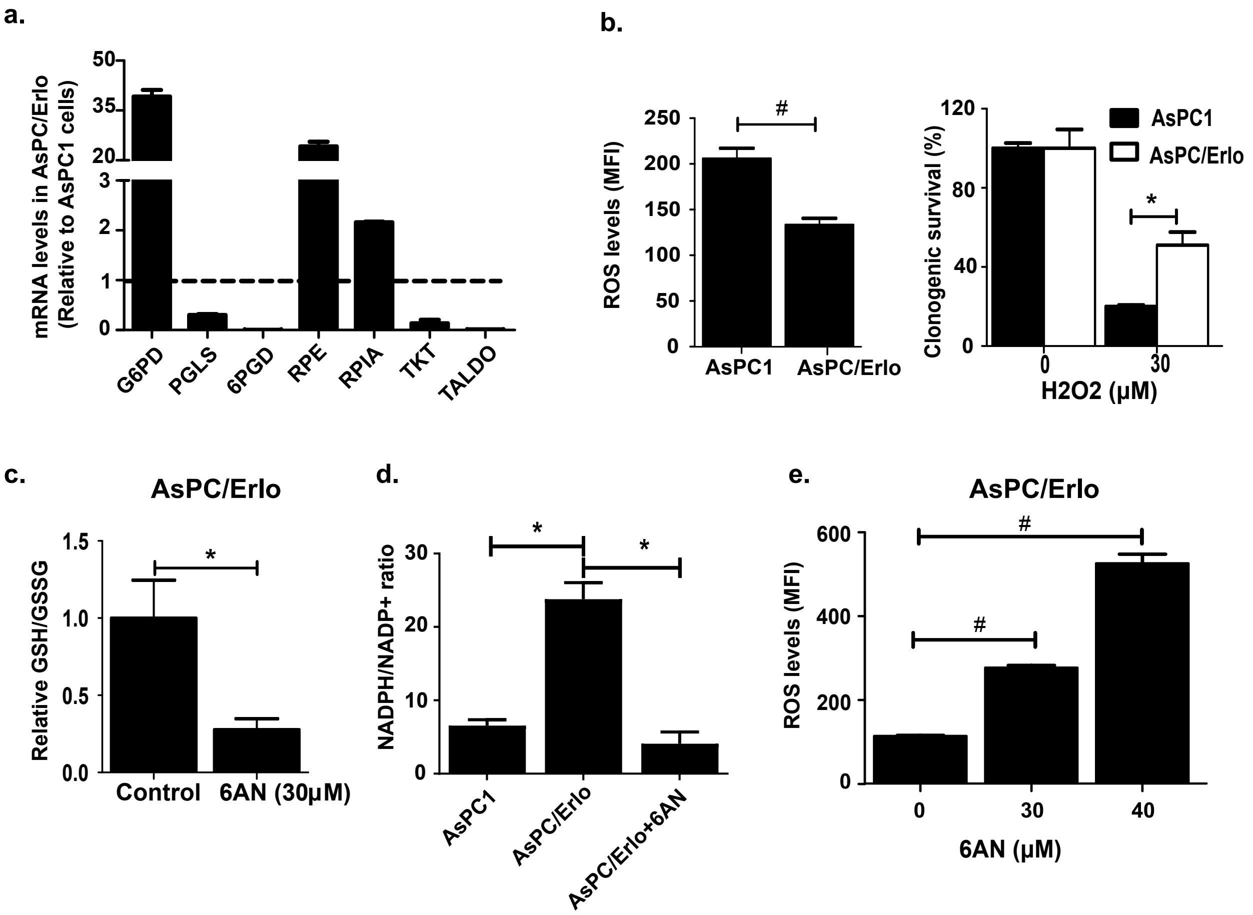


Supplemental S5: (a) Graph representing altered pentose phosphate pathway (PPP) enzyme mRNA levels in AsPC/Erlo cells as measured by real-time PCR analysis (n =3). (b) DCFDA stained cells were used to determine ROS levels in the cells (left). Cells treated with hydrogen peroxide (30 μM) for 10 minutes were analyzed for clonogenic survival (right) (n=3). (c) Reduced (GSH) and oxidized (GSSG) glutathione levels were analyzed using glutathione assay kit. Graph representing relative GSH/GSSG content in cells treated with 6AN for 48 hours (n= 3).
(d) NADPH/NADP levels were analyzed in indicated cells using commercial kit (n= 2). (e) The effect of 6AN on the induction of ROS was determined using DCFDA stained AsPC/Erlo cells (n= 4). Data presented as average ± SEM (*, $p < 0.05$, #, $p < 0.01$).
