# Supplementary material for: Metabolic plasticity imparts erlotinib-resistance in pancreatic cancer by upregulating glucose-6-phosphate dehydrogenase
Source: Cancer Metab. 2020 Sep 21;8:19. doi: 10.1186/s40170-020-00226-5 (PMC7507640; doi:10.1186/s40170-020-00226-5)
Supplement: Supplementary file 6 — Additional file 6. Supplemental S6: MTT assays were performed to determine the sensitivity of (a) MiaPaCa2 and MiaPaCa/Erlo, and (b) AsPC1 and AsPC/Erlo cells to metabolic inhibitors to pentose phosphate pathway (6AN), oxidative phosphorylation (Rotenone) and glycolysis (Iodoacetic acid). Data presented as average ± SEM (n= 3) (*, p < 0.05, #, p < 0.01). [file 40170_2020_226_MOESM6_ESM.pdf]

Supplemetal S6

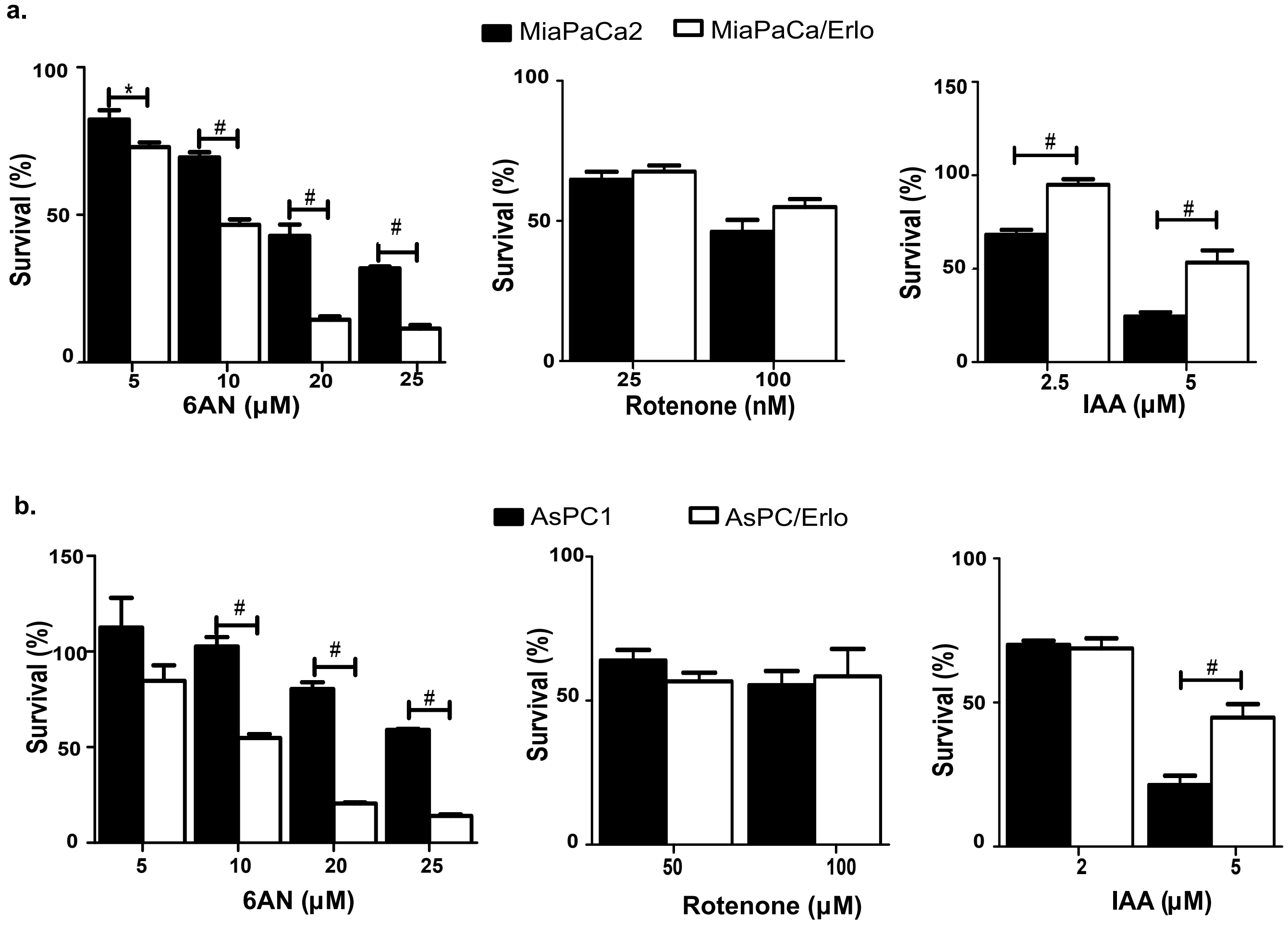

Supplemental S6: MTT assays were performed to determine the sensitivity of (a) MiaPaCa2 and MiaPaCa/Erlo, and (b) AsPC1 and AsPC/Erlo cells to metabolic inhibitors to pentose phosphate pathway (6AN), oxidative phosphorylation (Rotenone) and glycolysis (Iodoacetic acid). Data presented as average ± SEM (n= 3) (*, $p < 0.05$, #, $p < 0.01$).
