# Supplementary material for: Metabolic plasticity imparts erlotinib-resistance in pancreatic cancer by upregulating glucose-6-phosphate dehydrogenase
Source: Cancer Metab. 2020 Sep 21;8:19. doi: 10.1186/s40170-020-00226-5 (PMC7507640; doi:10.1186/s40170-020-00226-5)
Supplement: Supplementary file 7 — Additional file 7. Supplemental S7: (a) Effect of 6AN on sensitivity of AsPC1 and AsPC/Erlo cells to erlotinib (Erlo) was determined using clonogenic assay (n= 3). (b) Graph depicting sensitivity of pancreatic cancer cell lines (PANC-1, MiaPaCa2, AsPC1, and BxPC-3) to erlotinib (72-hour treatment) as measured by MTT assay (left). The effect of 6AN (48-hour treatment) on cytotoxicity of Erlotinib on PANC-1 cells was measured using clonogenic survival assay (n= 3). (c) Effect of 6AN on cell cycle was determined using propidium iodide stained cells (left). Immunoblot analysis were performed to determine alteration in cyclin levels by 48-hour 6AN treatment (right) (n= 4). (d) Effect of acute 6AN treatment (30 uM) on extracellular acidification rate of MiaPaCa2 and MiaPaCa/Erlo cells was assessed using Seahorse metabolic analyzer (n= 3). Data presented as average ± SEM (*, p < 0.05, #, p < 0.01). [file 40170_2020_226_MOESM7_ESM.pdf]

Supplemental S7

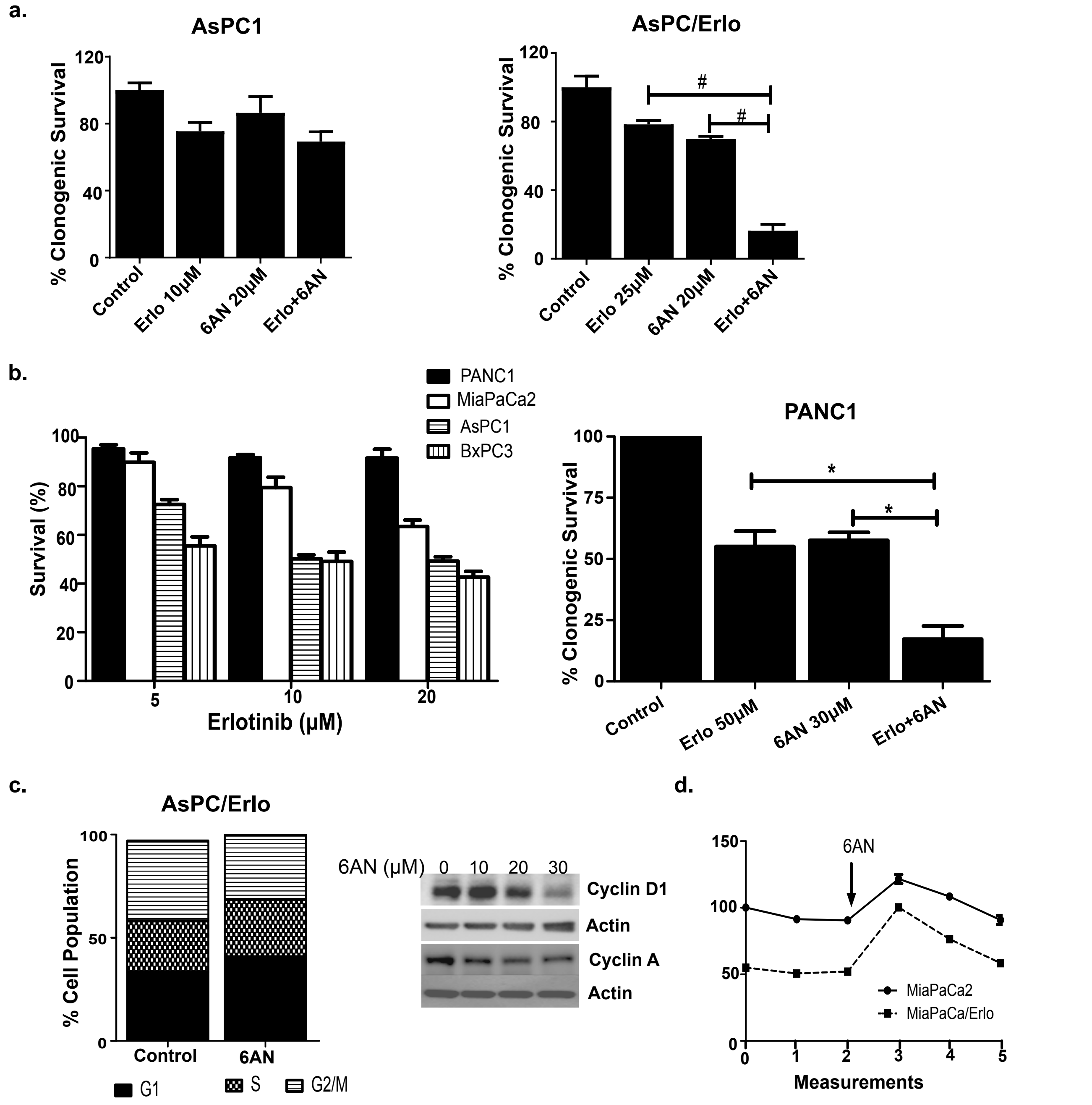

Supplemental S7: (a) Effect of 6AN on sensitivity of AsPC1 and AsPC/Erlo cells to erlotinib (Erlo) was determined using clonogenic assay (n= 3). (b) Graph depicting sensitivity of pancreatic cancer cell lines (PANC-1, MiaPaCa2, AsPC1, and BxPC-3) to erlotinib (72-hour treatment) as measured by MTT assay (left). The effect of 6AN (48-hour treatment) on cytotoxicity of Erlotinib on PANC-1 cells was measured using clonogenic survival assay (n= 3). (c) Effect of 6AN on cell cycle was determined using propidium iodide stained cells (left). Immunoblot analysis were performed to determine alteration in cyclin levels by 48-hour 6AN treatment (right) (n= 4). (d) Effect of acute 6AN treatment (30 uM) on extracellular acidification rate of MiaPaCa2 and MiaPaCa/Erlo cells was assessed using Seahorse metabolic analyzer (n= 3). Data presented as average ± SEM (*, $p < 0.05$, #, $p < 0.01$).
