# Supplementary material for: Metabolic plasticity imparts erlotinib-resistance in pancreatic cancer by upregulating glucose-6-phosphate dehydrogenase
Source: Cancer Metab. 2020 Sep 21;8:19. doi: 10.1186/s40170-020-00226-5 (PMC7507640; doi:10.1186/s40170-020-00226-5)
Supplement: Supplementary file 8 — Additional file 8. Supplemental S8: (a) Effect of G6PD knockdown (72 hours post siRNA transfection) on AsPC/Erlo cell cycle distribution was determined using flow cytometry (n =2). (b) Effect of G6PD knockdown on AsPC/Erlo cell sensitivity to erlotinib was determined using clonogenic assay (n= 3). (c) AsPC1 cells transfected with G6PD overexpression plasmid (G6PD/pRK5) were analyzed for their sensitivity to erlotinib using clonogenic survival assay. The results were compared with empty vector transfected AsPC/Erlo cells treated with erlotinib (n= 3). Data presented as average ± SEM (*, p < 0.05, #, p < 0.01. [file 40170_2020_226_MOESM8_ESM.pdf]

Supplmental S8

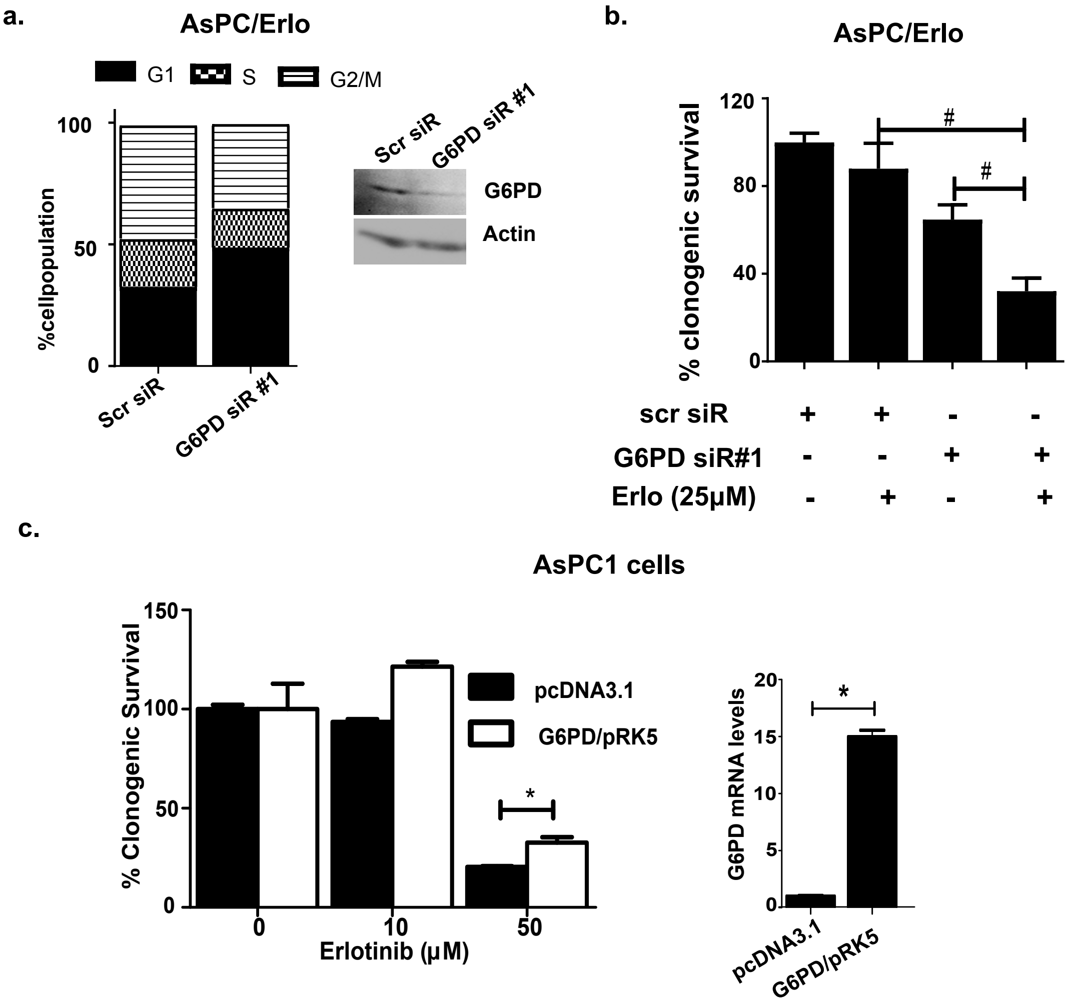

Supplemental S8: (a) Effect of G6PD knockdown (72 hours post siRNA transfection) on AsPC/Erlo cell cycle distribution was determined using flow cytometry (n =2). (b) Effect of G6PD knockdown on AsPC/Erlo cell sensitivity to erlotinib was determined using clonogenic assay (n= 3). (c) AsPC1 cells transfected with G6PD overexpression plasmid (G6PD/pRK5) were analyzed for their sensitivity to erlotinib using clonogenic survival assay. The results were compared with empty vector transfected AsPC/Erlo cells treated with erlotinib (n= 3). Data presented as average ± SEM (*, $p < 0.05$, #, $p < 0.01$).
