# Supplementary material for: Metabolic plasticity imparts erlotinib-resistance in pancreatic cancer by upregulating glucose-6-phosphate dehydrogenase
Source: Cancer Metab. 2020 Sep 21;8:19. doi: 10.1186/s40170-020-00226-5 (PMC7507640; doi:10.1186/s40170-020-00226-5)
Supplement: Supplementary file 9 — Additional file 9. Supplementary Information 1. [file 40170_2020_226_MOESM9_ESM.docx]

**Supplementary Information 1**

**Antibodies**

| **Protein** | **Cat#** | **Supplier** |
| --- | --- | --- |
| Erk1/2 | 9102 | Cell Signaling Technologies |
| p-Erk1/2 | 9101 | Cell Signaling Technologies |
| Akt1/2/3 | sc-8312 | Santa Cruz |
| p-Akt (ser473) | 9271 | Cell Signaling Technologies |
| mTOR | 2983 | Cell Signaling Technologies |
| p-mTOR | 2971 | Cell Signaling Technologies |
| Cyclin D1 | sc-718 | Santa Cruz |
| Cyclin A | sc-596 | Santa Cruz |
| Cyclin B1 | sc-245 | Santa Cruz |
| Cyclin E | sc-198 | Santa Cruz |
| Enolase | sc-271384 | Santa Cruz |
| LDHA | sc-137243 | Santa Cruz |
| PFK1 | sc-377346 | Santa Cruz |
| GPI | sc-365066 | Santa Cruz |
| c-myc | 9402 | Cell Signaling Technologies |
| G6PD | 12263 | Cell Signaling Technologies |
| Actin | AM4302 | Invitrogen |
| ID1 | sc-133104 | Santa Cruz |

**Real-time PCR Primers**

HPRT

FW: 5’ TTG GAA AGG GTG TTTA TTC CTC A 3’

RV: 5’ TCC AGC AGG TCA GCA AAG AA 3’

**Glycolysis Primers**

HK2 (Hexokinase 2)

FW: 5’ AGTGGAAGGCAGAGACGTTG 3’

RV: 5’ CAGTGCGAATGTCGTTGAGC 3’

LDHA (Lactate dehydrogenase A)

FW: 5’ GGATTCAGCCCGATTCCGTT3’

RV: 5’CCGTAAAGACCCTCTCAACCAC3’

Aldolase A

FW: 5’ TCCAGCTTCAACATGACCCAC 3’

RV: 5’ CCTAACTCTGTCTGGTGCTGG 3’

GPI (Glucose-6-phosphate isomerase)

FW: 5’ CGGTCAAACACACCCATCCT 3’

RV: 5’CTGTGTACCCCTTCCAGTCAC 3’

Enolase

FW: 5’ CTGTGCACTCAGAAACTGAACG 3’

RV: 5’GATGACATTGAACGCCGGGA 3’

PGK1 (Phosphoglycerate kinase 1)

FW: 5’ GCTGGACAAGCTGGACGTTA 3’

RV: 5’TCTGGGCCTACACAGTCCTT 3’

PGM1 (Phosphoglycerate mutase 1)

FW: 5’ TGGCCTCAGATTGCTCTCCT 3’

RV: 5’CGGTTAGACCCCCATAGTGC 3’

PFKM (phosphofructokinase, muscle)

FW: 5’ GTGCCCGTGTCTTCTTTGTC 3’

RV: 5’ CGTCCTTCTCGTTCCCGAAA 3’

PKM2 (Pyruvate Kinase Muscle 2)

FW: 5' CCGATCAGTGGAGCTGAAGAA 3’

RV: 5’ GCCACAGGATGTTCTCGTCA 3’

**Pentose Phosphate Pathway primers**

G6PD (Glucose 6 phosphate dehydrogenase)

FW: 5' CTACCGCATCGACCACTACC 3'

RV: 5' CCTGTTGGCAAATCTCAGCAC 3'

PGLS (6-phosphogluconolactonase)

FW: 5' GCCTCATCTCGGTGTTCTC 3'

RV: 5’ AGAGAAGATGCGTCCGGTAGA 3'

TKT (Transketolase)

FW: 5’ TTCCACACCATGCGCTACAA 3’

RV: 5’ CGGTGAAAGCTTGTTTCGGG 3

TALDO1 (Transaldolase 1)

FW: 5’ ACAGAAGTAGACGCAAGGCTC 3’

RV: 5’ ATTCGGTCCTTGCTGATCCC3’

6PGD (phosphogluconate dehydrogenase)

FW: 5’ ATATAGGGACACCACAAGACGG 3’

RV: 5’ CCGTTGTGCACCATCTTCAC 3’

RPE (ribulose-5-phosphate-3-epimerase)

FW: 5’ TGGGATGAAGGTTGGCCTTG 3’

RV: 5’ GGGTCCTCAACCAGTGAACC 3’

RPIA (ribose 5-phosphate isomerase A) PRIMER PAIR 2

FW: 5’ TGCTGGGAATTGGAAGTGGT 3’

RV: 5’ CGATCTCTGGGTGTCGATCC 3’
